# Myokine Response to High-Intensity Interval vs. Resistance Exercise: An Individual Approach

**DOI:** 10.3389/fphys.2018.01735

**Published:** 2018-12-03

**Authors:** Zihong He, Ye Tian, Pedro L. Valenzuela, Chuanye Huang, Jiexiu Zhao, Ping Hong, Zilin He, Shuhui Yin, Alejandro Lucia

**Affiliations:** ^1^Biology Center, China Institute of Sport Science, Beijing, China; ^2^Culture Development Center, General Administration of Sport of China, Beijing, China; ^3^Physiology Unit, Systems Biology Department, University of Alcalá, Alcalá de Henares, Spain; ^4^Graduate School, Shandong Sport University, Jinan, China; ^5^Winter Sports Administrative Center, General Administration of Sport of China, Beijing, China; ^6^Cardiovascular Department, Beijing Jian Gong Hospital, Beijing, China; ^7^Institute of Hepatobiliary Gastrointestinal Disease, The Rocket Force General Hospital of People’s Liberation Army (PLA), Beijing, China; ^8^Faculty of Sport Sciences, European University of Madrid, Villaviciosa de Odón, Spain; ^9^Instituto de Investigación Hospital 12 de Octubre (‘i+12’), Córdoba, Spain

**Keywords:** cytokines, metabolism, physical activity, training, responders

## Abstract

**Purpose:** This study aimed to compare the response to acute exercise of several myokines/hormones involved in metabolic function between two types of training sessions that are growing in popularity for their purported cardiometabolic benefits, high-intensity interval (HIIT) and resistance training (RT).

**Methods:** Seventeen healthy, non-athletic men (23 ± 3 years) participated in this cross-over study. They randomly performed a HIIT [with short (HIIT1) or long (HIIT2) intervals] or a RT session. The concentration of fibroblast-growth factor (FGF) 21, follistatin, ghrelin, interleukin-15, irisin, myostatin, and peptide YY was measured at baseline and 0, 1, 3, 24, 48, and 72 h post-exercise. An individual approach was adopted to determine the rate of responsiveness to each specific cytokine and training mode.

**Results:** A significant condition (session type) by time interaction (*p* = 0.004) effect was observed for FGF21, with RT eliciting a greater area under the curve (AUC) concentration than HIIT1 (*p* = 0.02). The AUC for follistatin was significantly greater after HIIT2 compared with RT (*p* = 0.02). Individual responsiveness to all session types ranged between 19 and 93% depending on the cytokine. However, most subjects (71–100%) responded positively for all cytokines (except for irisin, with only 53% of responders) after 1+ session type.

**Conclusion:** Except for FGF21, our results show no overall differences in the myokine response to HIIT or RT. A considerable individual variability was observed, with some subjects responding to some but not other training session types. Notwithstanding, most responded to at least one training session. Thus, it is mostly the individual response of each subject rather than general recommendations on type of training session (i.e., RT vs. HIIT or HIIT subtypes) that must be taken into consideration for maximizing cardiometabolic benefits in the context of personalized exercise prescription.

## Introduction

Regular physical exercise is an effective lifestyle intervention for the prevention and treatment of some of the most common non-communicable diseases, notably cardiometabolic conditions and many types of cancer (Fiuza-Luces et al., 2013, 2018; Ruiz-Casado et al., 2017). The numerous exercise benefits for cardiometabolic health and weight management are partly mediated by the production of cytokines or peptides in contracting muscles, the so-called myokines; which are released to the blood and exert endocrine or paracrine effects in other cells, tissues or organs (Pedersen and Febbraio, 2012; Fiuza-Luces et al., 2018).

Myokines and exercise-induced proteins/hormones in general play a role in a variety of physiological functions, including mainly muscle growth and metabolic homeostasis. Myostatin, the first described secreted muscle factor to fulfil the criteria of a myokine (Fiuza-Luces et al., 2013), is a negative regulator of muscle growth (Huang et al., 2011), whereas follistatin is a myostatin-binding protein that promotes skeletal muscle development through the activation of the mammalian target of rapamycin pathway (Winbanks et al., 2012). Besides their main function related to muscle plasticity, myostatin and follistatin also play a role in metabolism [i.e., reduction of body fat, improvement of glucose homeostasis and browning of white adipose tissue (WAT)] (Huang et al., 2011; Braga et al., 2014). Another important contraction-induced myokine is interleukin (IL)-15 (Fiuza-Luces et al., 2018), owing to its potential effects on metabolic homeostasis, through a decrease in WAT mass (Nielsen et al., 2008), and an enhancement of glucose tolerance (Kim H.J. et al., 2013) and glucose uptake by muscle tissue (Busquets et al., 2006). Other myokines that are also involved in metabolic homeostasis have been proposed as therapeutic targets for the management of obesity and its related complications. Notably, fibroblast growth factor (FGF) 21 is involved in glucose regulation and lipid utilization, and promotes weight loss and WAT browning (Woo et al., 2013; Fisher and Maratos-Flier, 2016). Irisin is also involved in the promotion of WAT browning with subsequent increases in thermogenesis (Elbelt et al., 2013; Perakakis et al., 2017). In turn, some hormones such as ghrelin (also known as the “hunger hormone”) and the peptide YY (PYY) provide metabolic benefits and promote weight management mainly through their influence on appetite, although they also exert a role on glucose and fatty acid homeostasis (Karra et al., 2009; Pradhan et al., 2013; Pinkney, 2014).

Although it is known that regular exercise might benefit cardiometabolic health through the cumulative effects of repeated episodes of exercise-induced increases in myokines or proteins/hormones (Ruiz-Casado et al., 2017), scarce evidence is available regarding which type of exercise session elicits a more robust effect on the release of these molecules. Further, although the existence of a wide inter-individual variability in the biological responses to a given exercise session and its importance for personalized exercise prescription is increasingly recognized, with some subjects achieving meaningful benefits (known as “responders”) and others showing no changes (“non-responders”) (Mann et al., 2014), most studies still report biological responses to exercise as group average.

Two training modes are gaining increasing popularity for health promotion and weight management. High intensity interval training (HIIT), which involves short bursts of high-intensity exercise (i.e., from less than 1 min to a maximum of 2–4 min) interspersed with short recovery periods is receiving considerable attention partly owing to the low time commitment it requires (<20 min per session) (Gibala and McGee, 2008). This training method has proven effective for the improvement of important health indicators such as cardiorespiratory fitness, metabolic biomarkers (e.g., of glucose control/insulin resistance) and body composition in both healthy and clinical populations (Gibala et al., 2012; Weston K.S. et al., 2014; Weston M. et al., 2014; Milanović et al., 2015; Wewege et al., 2017). Attending to the most recent annual survey of the American College of Sports Medicine, resistance training (RT) is also rapidly becoming one of the largest fitness trends (Thompson, 2017). RT has proven effective not only for the promotion of muscle mass/strength gains as traditionally thought (Borde et al., 2015), but also for reducing cardiometabolic risk factors such as obesity, insulin resistance or hypertension (Ibanez et al., 2005; Strasser et al., 2010).

The main purpose of this study was to compare the response of several myokines/hormones involved in cardiometabolic health to HIIT (two session types) vs. RT, with an analysis of both average and individual responses. Moreover, the effect of these training session types on resting metabolic rate (RMR) was also analyzed as a secondary endpoint given the influence of RMR on total daily energy expenditure and consequently on weight management and cardiometabolic health in general.

## Materials and Methods

### Participants

Seventeen male subjects participated in this study [(mean ± SD) age, 23 ± 2 years; body mass index, 22 ± 2 kg ⋅ m^2^]. Inclusion criteria were being healthy (i.e., free of any cardiovascular disease, diabetes or abnormal glucose tolerance, or any other acute/chronic disease) and performing no regular physical exercise (i.e., less than 20 min twice a week, or less than a total of 75 min during the week). Participants were required to maintain the same dietary habits during the study length, as well as to refrain from doing exercise, smoking, or drinking coffee or alcohol.

The experimental protocol was conducted in accordance with the Declaration of Helsinki and was approved by the Ethics Committee of the Chinese Institute of Sport Science. Before their inclusion in the study, subjects were informed of the objects and procedures and provided both verbal and written informed consent.

### Experimental Design

The study followed a cross-over, counterbalanced design. Each participant was assigned to perform a HIIT session with short (HIIT1) or long (HIIT2) intervals, or a RT session, in a randomized order, with a 7 days period between sessions.

One week before the first training session participants performed an incremental exercise test for VO_2max_ determination (see below). The day of the exercise sessions participants attended to the laboratory in the morning under fasting conditions, and we obtained blood samples and measured their RMR (baseline measures). Thereafter participants had the same standardized breakfast [one cup of soy milk, one egg, and two ∼50 g steamed stuffed buns (filled with pork)] and 2 h later they completed the prescribed training session. RMR was analyzed before (baseline) and 24, 48, and 72 h after each training session. All blood variables were analyzed before (baseline) and immediately after each training session, as well as 1, 3, 24, 48, and 72 h after each session.

### Training Sessions

Two common types of HIIT sessions were designed. HIIT1 consisted of two sets of six 30 s treadmill running bouts at 100% of the speed (V_max_) eliciting the VO_2max_ in the previous incremental text (see below), with 90 s of active recovery (50% of V_max_) between bouts and 4 min of passive recovery between sets. HIIT2 consisted of five 4 min bouts at 90% V_max_, with 4 min of active recovery (50% of V_max_) between bouts. Both sessions lasted approximately 45–50 min and were conducted on the same treadmill that was used for VO_2max_ determination (pulsar4.0; H/P/cosmos, Traunstein, Germany).

The RT session was based on the recommendations of the American College of Sports Medicine (American College of Sports Medicine [ACSM], 2009). Seven types of exercises targeting all the main muscle groups (back squat, bench press, barbell deadlift, barbell row, barbell military press, standing biceps curl, and sit-ups) were prescribed. Participants performed four sets of 8–10 repetitions at 70–75% of their one repetition maximum (1RM, which had been determined during a previous familiarization session) for all exercises except for sit-ups, for which they performed four sets of 20 repetitions without external weight (i.e., just their body weight). Subjects rested for 60–90 s between exercises and for 4 min between sets. Each session lasted ∼50 min.

### Measurements

#### Body Composition

Height and weight were measured to the nearest 0.1 cm and 0.1 kg, respectively, using a calibrated stadiometer and platform scale (JianminII, Beijing Xin Dong Hua Teng, Beijing, China). Body composition (fat and muscle mass, expressed in relative values) was determined by whole body dual energy X-ray absorptiometry scan (GE LUNAR DPX system, Madison, WI, United States).

#### Maximal Oxygen Uptake

The treadmill speed was initially set at 7 km ⋅ h^-1^ and thereafter was increased by 1 km ⋅ h^-1^ every 2 min until volitional exhaustion, while treadmill inclination was kept constant (at 0%). The test was deemed valid if at least three of the following criteria were met: (*i*) a plateau in VO_2_ was observed despite increasing exercise intensity; (ii) the subject was no longer able to maintain the required speed; (iii) the respiratory exchange ratio exceeded 1.10; and (iv) the age-predicted maximum heart rate (HR_max_, 220 *minus* age, in years) was achieved. Gas-exchange data were collected breath-by-breath during the tests with a metabolic cart (MetaMax 3B, Cortex, Biophysik, Germany).

#### Energy Expenditure During HIIT

Oxygen uptake (VO_2_) was analyzed with the aforementioned metabolic cart during HIIT sessions but not during RT sessions due to technical/logistic reasons. We also measured the heart rate (HR) response during HIIT sessions with a HR monitor (Polar RS400, Polar Electro, Kempele, Finland).

#### Resting Metabolic Rate

During measurements participants lied supine in a bed for 20 min (the first 3 min were discarded from the analyzes) in a room that had minimal light and noise and with ambient temperature maintained at 22 ± 1°C. VO_2_ was analyzed with the aforementioned metabolic cart and a variation < 25 ml ⋅ min^-1^ was used to determine that the collection was acceptable.

#### Blood Variables

Blood samples (10 ml each) were drawn from the antecubital vein and centrifuged (3000 × *g*) for 10 min. The serum was then kept at -80°C. Enzyme-linked immunosorbent assay (ELISA) was used for the analysis of the concentration of FGF21 [R&D Systems (Minneapolis, MN, United States), number: DF2100], follistatin (R&D Systems, number: DFN00), myostatin (R&D Systems, number: DGDF80), IL-15 (R&D Systems, number: 0707170149), irisin (Phoenix Pharmaceuticals (Burlingame, CA, United States), number: EK-067-29), acyl ghrelin (Phoenix Pharmaceuticals, number: EK-031-30), and PYY [Millipore (Burlington, MA, United States), number: EZHPYYT66K]. The standard curves were analyzed by double parallel tube. All the changes were analyzed as a percentage of baseline values. The peak concentration and the area under the curve (AUC) displayed by the concentration-time data (trapezoid rule) were analyzed for each molecule.

### Statistical Analysis

All data analyzed are available as Supplementary Material. Data are presented as mean ± SD. The normal distribution (Shapiro–Wilk test) and homoscedasticity (Levene’s test) of the data were checked before any statistical treatment. Student’s paired *t-*tests were conducted to analyze the differences in energy expenditure during HIIT1 and HIIT2. A two-factor [condition (HIIT1, HIIT2, RT) and time] repeated-measures ANOVA was used to compare the response over time of the blood variables and RMR between the three types of training sessions (HIIT1, HIIT2, RT). A Greenhouse–Geisser correction was applied when Mauchly’s Test of Sphericity was violated. One-way repeated measures ANOVA was used to analyze differences between training sessions (HIIT1, HIIT2, RT) in peak levels and AUC for each blood variable. All statistical analyses were conducted using a statistical software package (SPSS 23.0, United States) setting the significance level at α = 0.05.

The rate of responders was calculated for each blood variable and for RMR. Responsiveness was defined as positive changes that exceeded two times the typical error of measurement (TE) (Hopkins, 2000). The TE was calculated for each variable as the standard error of within-subjects standard deviation for all baseline measures (three measurements for each variable) (Hopkins, 2015). This value was multiplied by 2 and expressed as a percentage of the condition’s mean. The responsiveness threshold (i.e., 2 × TE expressed as a percentage of the three measures’ mean) for FGF21, follistatin, myostatin, IL-15, irisin, ghrelin and PYY was 95.5, 72.7, 36.9, 139.5, 37.7, 77.2, and 47.9%, respectively, whereas it equaled 15.9% for RMR. Student’s unpaired *t*-tests were performed to analyze differences in body composition and VO_2_max between responders and non-responders. We used Fishers’ exact test to compare the rate of responders for each blood variable between training session types (3 [HIIT1, HIIT2, RT] × 2 [responder vs. non-responder] contingency table). When a significant *p*-value (*p <* 0.05) was observed, we performed the test using 2 × 2 contingency tables to determine differences between specific training session types.

## Results

Subjects’ VO_2max_ averaged 49 ± 5 ml ⋅ kg ⋅ min^-1^. Their total fat and muscle mass averaged 10 ± 4 and 57 ± 5 kg, respectively. All participants completed the prescribed training sessions at the required intensities.

### Energy Expenditure During HIIT Sessions

HIIT1 induced a higher mean heart rate than HIIT2 (88 ± 4 vs. 83 ± 5% of HR_max_, respectively; *p* < 0.001), as well as a higher mean energy aerobic expenditure (82 ± 6 vs. 64 ± 5% of VO_2max_, respectively; *p* < 0.001 or 826 ± 110 vs. 641 ± 72 kcal, respectively; *p* < 0.001). The differences in caloric expenditure were mostly due to a higher contribution of carbohydrate metabolism in HIIT1 (154 ± 31 vs. 119 ± 16 g, respectively; *p* < 0.001), with no significant differences being observed for fat (11 ± 4 vs. and 10 ± 4 g, *p* = 0.540) or protein oxidation (22 ± 6 vs. 14 ± 2g, *p* = 0.062).

### Resting Metabolic Rate

**FIGURE 1:**
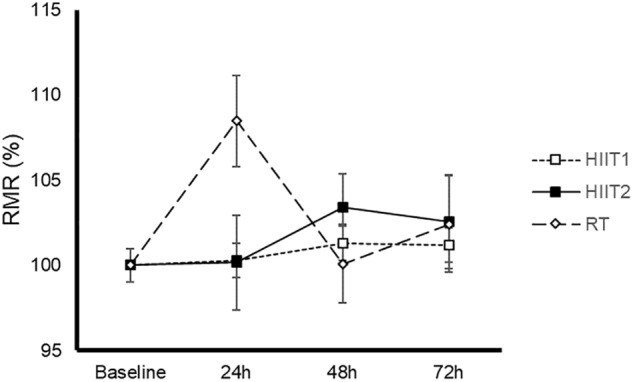
Time-course of resting metabolic rate (RMR) after a session of high-intensity interval training with short (HIIT1) or long intervals (HIIT2), or resistance training (RT). Data are mean ± standard error. No significant time (*p* = 0.164) or condition (HIIT1, HHT2 or RT) by time interaction effect (*p* = 0.058) was found. Abbreviations: HIIT1, high intensity interval training session with short intervals; HIIT2, high intensity interval training session with long intervals; RT, resistance training.

We found no significant time (*p* = 0.164) or condition by time interaction effect (*p* = 0.058) (Figure 1). Only 6, 12, and 24% of the subjects could be considered responders attending to their RMR after HIIT1, HIIT2, and RT, respectively.

### Blood Variables

**FIGURE 2:**
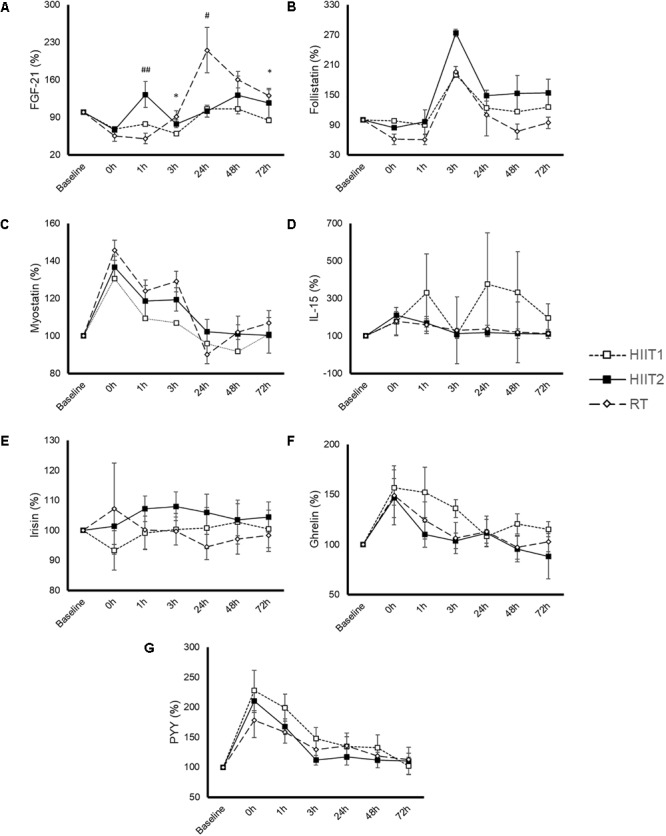
Time-course response of fibroblast growth factor 21 (FGF21) **(A)**, follistatin **(B)**, myostatin **(C)**, interleukin (IL)-15 **(D)**, irisin **(E)**, ghrelin **(F),** and peptide YY (PYY) **(G)** to a session of high-intensity interval training with short (HIIT1) or long intervals (HIIT2), or resistance training (RT). All molecules were measured in 17 subjects except for IL-15 and PYY, which were measured in 9 and 14 subjects, respectively. Data are mean ± standard error. A significant (*p* < 0.05) time effect was observed for all variables except for irisin. A significant condition by time interaction was just observed for FGF21 (*p* = 0.004).

**FIGURE 3:**
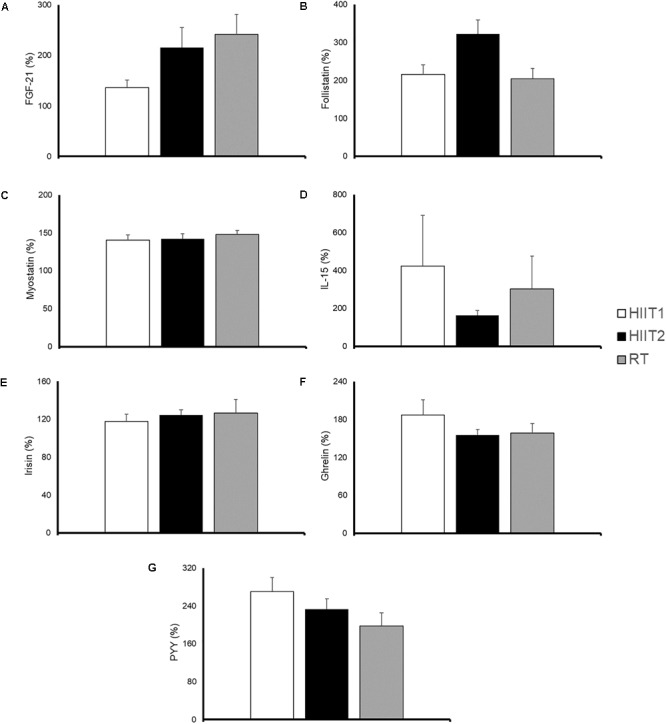
Peak concentration of fibroblast growth factor-21 (FGF-21) **(A)**, follistatin **(B)**, myostatin **(C)**, interleukin (IL)-15 **(D)**, irisin **(E)**, ghrelin **(F),** and peptide YY (PYY) **(G)** in response to a session of high-intensity interval training with short (HIIT1) or long intervals (HIIT2), or resistance training (RT). All molecules were measured in 17 subjects except for IL-15 and PYY, which were measured in 9 and 14 subjects, respectively. Data are mean ± standard error. There were no significant differences between conditions.

**FIGURE 4:**
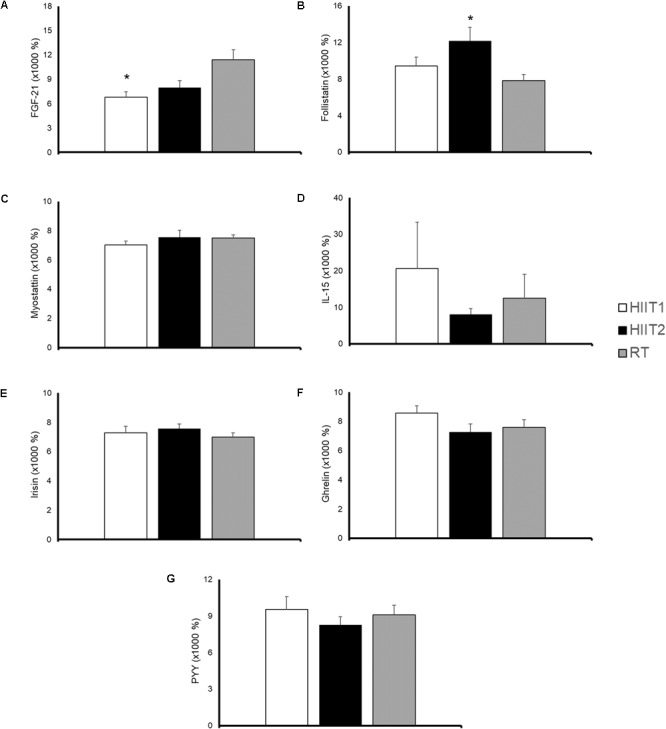
Area under the curve (calculated from concentration-time data from 0 to 72 h post-exercise) of fibroblast growth factor-21 (FGF-21) **(A)**, follistatin **(B)**, myostatin **(C)**, interleukin (IL)-15 **(D)**, irisin **(E)**, ghrelin **(F)**, and peptide YY **(G)** in response to a session of high-intensity interval training with short (HIIT1) or long intervals (HIIT2), or a resistance training (RT) session. All molecules were measured in 17 subjects except for IL-15 and PYY, which were measured in 9 and 14 subjects, respectively. Data are mean ± standard error. Significantly different from RT: ^∗^*p* < 0.05.

**FIGURE 5:**
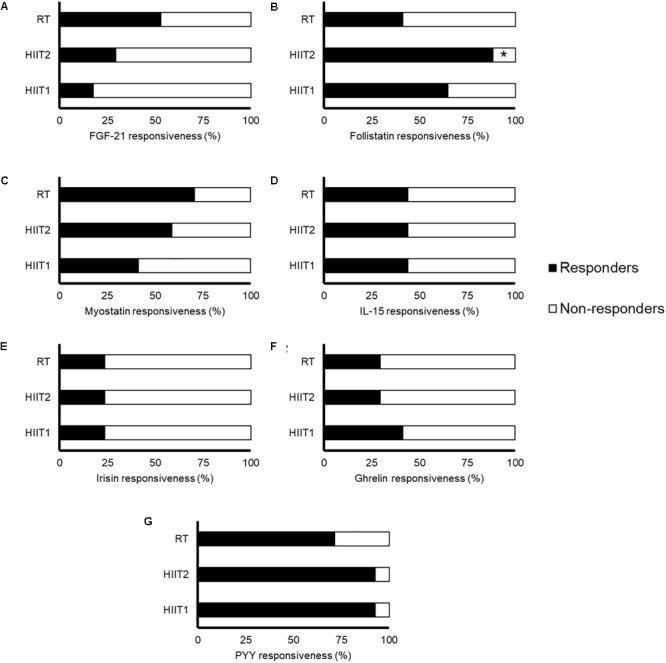
Rate of responsiveness of fibroblast growth factor-21 (FGF-21) **(A)**, follistatin **(B)**, myostatin **(C)**, interleukin (IL)-15 **(D)**, irisin **(E)**, ghrelin **(F)**, and peptide YY **(G)** in response to a session of high-intensity interval training with short (HIIT1) or long intervals (HIIT2), or a resistance training (RT) session. Responsiveness was determined as a positive change greater than the smallest worthwhile change (calculated for each molecule as twice the typical error of measurement and expressed as a percentage). All molecules were measured in 17 subjects except for IL-15 and PYY, which were measured in 9 and 14 subjects, respectively. Significantly different from RT: ^∗^*p* < 0.05.

**FIGURE 6:**
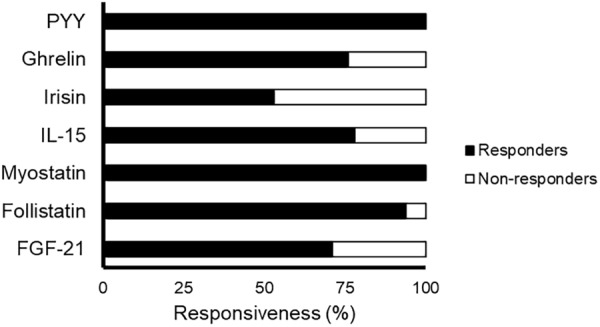
Rate of responsiveness of the blood variables to exercise independently of the training mode. Responsiveness was determined as a positive change greater than the smallest worthwhile change (calculated for each molecule as twice the typical error of measurement and expressed as a percentage). All molecules were measured in 17 subjects except for IL-15 and PYY, which were measured in 9 and 14 subjects, respectively. Abbreviations: FGF21, fibroblast growth factor 21; HIIT1, High intensity interval training session with short intervals; HIIT2, High intensity interval training session with long intervals; IL-15, interleukin-15; PYY, peptide YY; RT, resistance training.

The time course and peak and AUC concentrations of each myokine/hormone in response to exercise are presented in Figures 2–4, respectively. The individual response of each subject to the different training sessions is presented in Table 1. The rate of responsiveness for each molecule attending to the type of training session performed and independently of the training session mode is presented in Figures 5, 6, respectively.

**Table 1:** Individual biological response to the different training session types.

	FGF21			Follistatin			Myostatin			IL-15			Irisin			Ghrelin			PYY		
Subject	HIIT1	HIIT2	RT	HIIT1	HIIT2	RT	HIIT1	HIIT2	RT	HIIT1	HIIT2	RT	HIIT1	HIIT2	RT	HIIT1	HIIT2	RT	HIIT1	HIIT2	RT
1		+	+	+	+	+	+	+	+	+	+		+			+			+	+	+
2				+	+	+		+	+	NA	NA	NA	+						+	+	
3			+	+	+	+		+	+	NA	NA	NA						+	+	+	+
4				+	+	+			+	NA	NA	NA				+			+	+	+
5							+			NA	NA	NA							+	+	+
6	+		+	+	+				+	NA	NA	NA				+		+	+	+	+
7	+			+			+	+		+				+		+			+	+	
8					+	+		+	+		+		+				+		+	+	
9		+			+	+	+	+	+			+					+		+		+
10			+		+	+		+		+		+	+		+				+	+	
11			+		+				+		+	+								+	+
12			+	+	+				+							+			+	+	+
13	+	+		+	+			+	+	+	+	+		+	+			+	+	+	+
14			+	+	+		+	+	+					+		+	+		NA	NA	NA
15				+	+		+		+	NA	NA	NA					+		NA	NA	NA
16		+	+	+	+		+			NA	NA	NA			+		+	+	NA	NA	NA
17		+	+		+			+		NA	NA	NA		+	+	+		+	+	+	+

*Responsiveness (indicated as “+”) was determined as a positive change greater than the smallest worthwhile change (calculated for each cytokine as twice the typical error of measurement and expressed as a percentage). Abbreviations: FGF21, fibroblast growth factor; HIIT1, high intensity interval training session with short intervals; HIIT2, high intensity interval training session with long intervals; IL-15, interleukin-15; N/A, not available; PYY, peptide YY; RT, resistance training.*

#### Fibroblast Growth Factor 21

A significant time (*p* = 0.000) and condition by time interaction (*p* = 0.004) effect was observed for FGF21 (Figure 2A). Both HIIT1 and HIIT2 induced a significant increase in FGF21 levels at 3 (*p* = 0.002) and 0 h (*p* < 0.001) post-exercise, whereas the values of this protein increased above baseline values 48 h after RT (*p* = 0.025). A significantly higher AUC concentration was observed for RT vs. HIIT1 (*p* = 0.020), but not vs. HIIT 2 (*p* = 0.122) (Figure 4A).

Most subjects (>∼50%) could be considered non-responders for the FGF21 response to all types of training sessions, with no significant differences between sessions (*p* = 0.115) (Figure 5A). However, only 5 subjects (29%) did not increase their FGF21 levels in response to at least one of the three different training session types (Table 1 and Figure 6). No differences in fat mass (*p* = 0.765), muscle mass (*p* = 0.353) or VO_2max_ (*p* = 0.182) were observed between responders and non-responders for FGF21 (data not shown).

#### Follistatin

A significant time (*p* < 0.001) but not condition by time interaction (*p* = 0.176) was observed for follistatin (Figure 2B). RT yielded an almost significantly lower peak follistatin concentration than HIIT1 (*p* = 0.056) and HIIT2 (*p* = 0.085) (Figure 3B), and a significantly lower AUC than HIIT 2 (*p* = 0.016) (Figure 4B).

There was a significant relationship between training session type and responsiveness to follistatin (*p* = 0.019). Most subjects could be considered responders to follistatin after the HIIT sessions (Figure 5B). In contrast, 59% of participants did not show an increase in follistatin levels after RT, being this rate significantly lower than that observed with HIIT2 (*p* = 0.010) but not HIIT1 (*p* = 0.303) (Figure 5B). Only one subject (6%) did not show an increase in follistatin levels in response to any of the three training session types (Table 1 and Figure 6).

#### Myostatin

A significant time (*p <* 0.001) but not condition by time interaction effect (*p* = 0.280) was observed for myostatin (Figure 2C). No differences were observed between training session types in the peak (Figure 3C) or AUC concentration (Figure 4C) of this myokine.

Similar rates of responders were observed between conditions (*p* = 0.260) (Figure 5C), and although the rate of responders was overall low attending to each specific type of session (40–70%), all participants showed an increase in myostatin levels after at least one training session type (Table 1 and Figure 6).

#### Interleukin-15

Eight participants presented IL-15 concentrations below the minimum detection levels and thus their results could not be analyzed (total n for analyses = 9). No significant time (*p* = 0.373) or condition by time interaction (*p* = 0.324) was observed for this myokine (Figure 2D), with no differences between conditions in peak (Figure 3D) or AUC concentration (Figure 4D).

Approximately half of the participants (44%) could be considered non-responders for IL-15 attending to each specific training mode, with no differences between them (*p* = 1.0) (Figure 5D). However, all but two subjects (78%) responded positively after at least one type of training session (Table 1 and Figure 6).

#### Irisin

No significant time (*p* = 0.892) or condition by time interaction effect (*p* = 0.543) was observed for irisin levels (Figure 2E), with no differences between conditions in peak (Figure 3E) or AUC concentration (Figure 4E).

The rate of responders to irisin for all training session types was overall low (>75%), with no significant differences between them (*p* = 1.00) (Figure 5E). Eight subjects (47%) did not exhibit an increase in irisin levels in response to any of the three types of training session (Table 1 and Figure 6). No differences in fat mass (*p* = 0.721), muscle mass (*p* = 0.250) or VO_2max_ (*p* = 0.156) were observed between responders and non-responders for irisin (data not shown).

#### Ghrelin

A significant time (*p <* 0.001) but not condition by time interaction (*p* = 0.286) was observed for ghrelin (Figure 2F). No significant differences (*p* > 0.05) were observed between conditions for ghrelin peak (Figure 3F) or AUC concentration (Figure 4F).

The rate of responders for all three training modes was low (<50%), with no differences between conditions (*p* = 0.808) (Figure 5F). However, only four subjects (24%) did not show an increase in ghrelin levels in response to any type of session (Table 1 and Figure 6). No differences in fat mass (*p* = 0.702), muscle mass (*p* = 0.911) or VO_2max_ (*p* = 0.478) were observed between responders and non-responders for ghrelin (data not shown).

#### Peptide YY

Three participants presented a PYY concentration below the minimum detection levels and thus their results could not be analyzed (total n for analyses = 14). A significant time (*p <* 0.001) but not condition by time interaction (*p* = 0.452) was observed for PYY (Figure 2G). There were no differences between conditions in peak (Figure 3G) or AUC (Figure 4G) PYY concentration.

Almost all the subjects could be considered responders to PYY after the HIIT sessions, with no significant differences between conditions (*p* = 0.326) (Figure 5G). All the subjects increased their PYY levels in response to at least two training modes (Table 1 and Figure 6).

## Discussion

We have measured the exercise response of several myokines/hormones that are involved in metabolic regulation and weight management after a session of RT or HIIT, both of which are gaining popularity for their purported cardiometabolic benefits. In addition, we assessed inter-individual variability. Thus, our study is of potential relevance in the context of personalized exercise prescription for maximizing the cardiometabolic benefits of this crucial lifestyle intervention. In this context, the main finding of this study was the great inter-individual variability observed in the acute cytokine response to different types of training sessions, with the rate of responsiveness to each session ranging between 19 and 93% depending on the analyzed molecule. However, our individual approach shows that most subjects (71–100%) showed a positive response of all blood variables to at least one session type (except for irisin, with only 53% of responders). By contrast, the majority of subjects were non-responders for changes in RMR in the days following the exercise sessions, and no differences in the time course of RMR post-exercise were found between the three conditions. Given the beneficial role of exercise-induced factors (notably, myokines) for cardiometabolic health (Pedersen, 2011; Fiuza-Luces et al., 2018), our results suggest that training prescription should include a variety of stimuli including both RT and HIIT sessions in order to obtain the greatest benefits from exercise.

The myokines/hormones analyzed here play a major role in muscle growth and/or metabolism. Interestingly, all three session types resulted in a transient (up to 3 h after exercise) increase in myostatin, a transforming growth factor (TGF) β family member that acts as a negative regulator of muscle growth, followed by a subsequent gradual decrease, reaching again approximately baseline levels 72 h post-exercise. The effects of acute exercise on myostatin remain unclear, with some studies finding a down-regulation of myostatin mRNA expression after different types of exercise (Louis et al., 2007; Hittel et al., 2010; Lundberg et al., 2012) but others failing to find such changes (Jensky et al., 2010; MacKenzie et al., 2013). Although MacKenzie et al. (2013) found a decrease in myostatin transcriptional activity after resistance exercise, the exercise stimulus also activated Notch, an TGFβ inhibitor. The authors concluded that despite the acute increase in myostatin expression, the inhibition of its transcriptional activity might contribute to exercise-induced skeletal muscle hypertrophy (MacKenzie et al., 2013).

In the present study the increase in myostatin concentration upon exercise termination occurred concomitantly with an increase 3 h post-exercise in follistatin, a myostatin-binding protein involved in skeletal muscle development, energy metabolism and WAT browning (Winbanks et al., 2012; Braga et al., 2014). Previous research has demonstrated that this protein is released into the circulation in response to exercise, which could have potential effects on muscle hypertrophy and metabolism (Hansen et al., 2011). Our results show that the increase in follistatin was more marked after HIIT1 and especially HIIT2 compared to RT, with only one subject (6%) not responding positively to any of the HIIT sessions. Therefore, HIIT, especially if including long intervals, appears as the most effective strategy to increase follistatin concentration.

FGF21 has been proposed as a myokine induced by the PI3K–AKT pathway that plays important metabolic roles (Lee et al., 2012). Thus, FGF21 protects muscle tissue against insulin resistance (Lee et al., 2012), augments brown fat thermogenesis in concert with irisin (Lee et al., 2014), and is related to weight loss and WAT browning (Woo et al., 2013; Fisher and Maratos-Flier, 2016). We found an overall increase in the levels of FGF21 after exercise, which is in agreement with previous research (Kim K.H. et al., 2013; Tanimura et al., 2016). However, although all exercise types induced increases in FGF21, an interesting finding was that the increase tended to be greater after RT than after the HIIT sessions, remaining elevated above baseline values even 48 h after the former. Given the potential of FGF21 as a therapeutic target against metabolic disorders such as obesity and diabetes (Giralt et al., 2015; Strowski, 2017), these results could have promising clinical implications, being RT the most recommended training mode for the stimulation of FGF21.

In contrast, no consistent increases in IL-15 levels were observed after any of the training session types. Increased circulating levels of this myokine have been previously observed in young subjects immediately and up to 24 h after a RT session (Riechman, 2004; Pérez-López et al., 2018). A strong inverse relationship has been reported between the serum IL-15 response to exercise and the training volume and time under tension during a session, suggesting that prolonged muscle activation (such as that possibly elicited here by the 50 min session, which included a total of 28 sets of different whole-body exercises) might attenuate the release of this myokine (Pérez-López et al., 2018). Thus, although the present study does not support a stimulating role of the applied exercise training sessions on IL-15 secretion, other training types (i.e., brief but intense RT sessions) might increase the levels of this anti-catabolic/anti-obesogenic myokine. More research is, however, needed to elucidate how circulating IL-15 might influence skeletal muscle and adipose tissue mass in humans.

Another interesting finding of our study was the low rate (<25%) of responders for irisin, with no significant time or condition by time effect been found for this protein. Great attention has been given in recent years to the potential of irisin for the prevention and treatment of obesity and its related complications due to its purported role in WAT browning and energy expenditure (Pedersen and Febbraio, 2012; Elbelt et al., 2013). Previous research has shown an overall increase in circulating irisin levels after acute exercise, with the effect being independent of the type of exercise training session (resistance vs. aerobic training) but fitness level being the best response predictor (i.e., being fit is associated with a ∼twofold increase in post-exercise irisin) (see Fox et al., 2018 for a review). In this regard, we found no differences in VO_2max_ between responders and non-responders, but no data were available regarding participants’ muscle strength or specific training background, which might have also conditioned the irisin response. Notwithstanding, controversy now exists around irisin: concerns have been raised regarding inconsistencies between animal and human data (Raschke et al., 2013) and methodological issues (such as potential cross-reactivity of the commercially available anti-irisin antibodies with other proteins) (Albrecht et al., 2015). Our results add further controversy to this topic, as a very low rate of responders for all three different training modes was observed. Therefore, further research should address the actual effect of exercise on irisin concentrations and the physiological consequences of these increases.

Lastly, we observed an acute increase in the appetite-regulating hormone PYY in all the subjects upon exercise termination in response to at least one type of session, with most subjects (70%) increasing their PYY levels in response to each specific training mode. In line with these results, meta-analytical evidence concluded that exercise influences appetite by increasing the levels of hormones such as PYY, pancreatic polypeptide or glucagon-like peptide 1, which suppress food intake (Schubert et al., 2014). The influence of acute exercise on appetite might also be mediated, at least partly, by a reduction of acylated ghrelin levels. However, whereas some studies observed increased ghrelin levels in response to exercise (Broom et al., 2008; King et al., 2017), others reported opposite findings (Jürimäe et al., 2007; Mackelvie et al., 2007). Our results show that only a few participants (30–40%) presented acute increases in ghrelin levels in response to each of the three training sessions. However, a great percentage of the subjects (76%) showed an acute increase in response in ghrelin to at least one type of training session. The individual variability observed in our and other studies (King et al., 2017) might contribute to the existing controversy on the effects of exercise on ghrelin levels.

Some methodological limitations must be noted. Although all sessions had approximately the same duration (∼45–50 min), they were not matched for external or internal load (as reflected by the different energy expenditures). In addition, due to budget constraints we did not measure some important myokines such as interleukin-6 or brain-derived neurotrophic factor. Moreover, the time points at which blood samples were taken were chosen attending to practical/feasibility reasons rather than to an *a priori* analysis of the time-course of each myokine in response to exercise. Thus, we cannot rule out the existence of potential differences between sessions in the myokine response at time points others than those chosen for the present study. Lastly, the responsiveness threshold (2 × TE) takes into account the random error, that is, the variability provoked by the technical error of measurement and the biological day-to-day changes of each variable. Notwithstanding, we cannot discern if acute changes of a magnitude greater than this threshold actually translate into clinically meaningful benefits/adaptations. It is also important to highlight that the results obtained here might not be generalized to other populations such as overweight or elderly subjects, in whom the production of exercise-induced myokines would be maybe expected to induce greater health benefits than in healthy young adults as those assessed here. Therefore, future research should address the specific response among different population segments. More research is also warranted analyzing the myokine response to different training modes or loads for a given type of training (e.g., RT with varying training intensities or volumes). Finally, it must be emphasized that acute myokine responses as those studied here are not necessarily linked with actual chronic adaptations to training (Barros et al., 2017).

## Conclusion

The present study shows an overall higher FGF21 response to RT than to HIIT in healthy young subjects. In turn, a higher follistatin response was observed with HIIT (at least with long intervals, i.e., HIIIT2) than with RT. Most important, there was a considerable inter-individual variability in the response of the different cytokines irrespective of the type of exercise session. Notwithstanding, most subjects responded positively to at least one training mode except irisin, for which half of the participants showed no response. Given the involvement of the studied myokines and hormones on cardiometabolic health, our results suggest that, to obtain the greatest benefits, training prescription should be individualized in order to provide the necessary stimulus to each subject.

## Author Contributions

ZihH, YT, CH, JZ, PH, ZilH, and SY conceived the study and performed the experiments. PV and AL analyzed the data and drafted the manuscript. All authors significantly contributed to the final version of the manuscript.

## Conflict of Interest Statement

The authors declare thatthe research was conducted in the absence of any commercial or financial relationships that could be construed as a potential conflict of interest.
